# Pediatric out-of-hospital cardiopulmonary resuscitation by helicopter emergency medical service, does it has added value compared to regular emergency medical service?

**DOI:** 10.1007/s00068-017-0815-5

**Published:** 2017-07-15

**Authors:** X. R. J. Moors, K. Rijs, D. Den Hartog, R. J. Stolker

**Affiliations:** 1 Department of Pediatric Anesthesiology, Erasmus MC, University Medical Center Rotterdam-Sophia Children’s Hospital, P.O. Box 2060, 3015 CN Rotterdam, The Netherlands; 2 HEMS, Erasmus University Medical Center, Rotterdam, The Netherlands; 3 Department of Surgery-Traumatology, Erasmus University Medical Center, Rotterdam, The Netherlands

**Keywords:** EMS, HEMS, Pediatric, Survival, CPR, Prehospital

## Abstract

**Purpose:**

To determine the outcome of out-of-hospital (OOH) cardiopulmonary resuscitation (CPR) and the advanced life support (ALS) procedures provided in pediatrics by the Rotterdam Helicopter Emergency Medical Service (HEMS)

**Methods:**

Retrospective evaluation of all pediatric (0–17 years) OOH cardiopulmonary arrests within a 6-year period and attended by the Rotterdam HEMS team.

**Results:**

There were 201 OOH CPRs from October 2008 until October 2014. Endotracheal intubation was performed in 164 cases and done by HEMS in 104 patients (63%), intraosseous/intravenous cannulation 43/27 times, and additional medication given by HEMS in 70 patients (35%). The overall survival rate for OOH CPR was 15%, but in trauma was low. Twenty-seven of the 29 pediatric patients who survived until discharge are neurological well. Although the Dutch nationwide ambulance protocol states intubation, intravenous, or intraosseal excess and medication, in many patients, only HEMS provided additional ALS care.

**Conclusion:**

The HEMS brings essential medical expertise in the field not provided by regular emergency medical service. HEMS provide a significant quantity of procedures, obviously needed by the OOH CPR of a pediatric patient.

## Introduction

The Helicopter Emergency Medical Service (HEMS) was introduced in The Netherlands in 1995, enabling the delivery of a medical team to the scene in addition to the regular ambulance service. A HEMS team consists of a physician (board-certified anesthesiologist or trauma surgeon), a specialized nurse [Paramedic or Registered Nurse from the Emergency Department (ED)] and a helicopter pilot.

The Emergency Medical Service (EMS) protocol in The Netherlands is a nationwide protocol with precise description of procedures to follow, but the ambulance crew is limited in expertise and experience in vitally compromised children [1, 2]. When HEMS became operational, EMS frequently secondary asked for assistance in stabilizing vitally compromised children. After a few years, it became protocol to activate HEMS primarily in vitally compromised children. Prehospital data concerning pediatric EMS and/or pediatric HEMS in The Netherlands are lacking and it is difficult to extrapolate research done in other countries due to the differences in their HEMS and EMS organizations and HEMS dispatch criteria. Previous studies show a low survival rate in pediatric out-of hospital cardio pulmonary resuscitation (OOH CPR) [3–7]. This study was done to assess the survival rate and outcome in OOH CPR in pediatric patients treated by the Rotterdam HEMS. It is difficult to measure the expertise that leads to additional care provided by HEMS. To do so, we wanted to evaluate the medical interventions done either by the HEMS or by the EMS and to examine how often the HEMS provided this additional medical care, which was or could not be provided by the EMS.

## Methods

We performed a retrospective analysis of a database in which every patient treated by Rotterdam HEMS is registered. Only patients under the age of 18 on the day of the emergency call were included in the period October first 2008 until October first 2014. We selected all consecutive patients who underwent OOH CPR.

We considered an OOH cardio pulmonary arrest when EMS and/or HEMS objectified the indication of OOH CPR by clinical assessment, because it is difficult for a non-professional to assess if an arterial pulse is present in a pediatric patient [8]. The Pediatric Basic Life Support (PBLS) guidelines also advise PBLS providers when in doubt to start CPR. This study included pediatric patients by PBLS providers (police or fire brigade) only when an automatic external defibrillator (AED) gave a shock, this to avoid pediatric patients with poor circulation but with cardiac output.

Patients primarily treated in other hospitals and then transferred to our hospital were excluded.

After identifying all pediatric OOH CPR cases, we analyzed all ALS procedures, done by EMS and/or HEMS and the outcome of every case. We divided the groups in trauma, drowning, CPR at birth and non-trauma. The non-trauma group includes patients who do not fit in one of the other groups, for example, septic patients or patients with cardiomyopathy, etc. The unknown group consists of patients, due to missing data, and we did not know which group they belonged to.

Our primary outcome parameters were survival and success rate of ALS procedures, such as intubation, venous access, and intraosseal access. Secondary predictors were cause of OOH CPR and first rhythm.

## Results

A total of 201 pediatric OOH CPRs were selected in the database (Table 1).

**Table 1:** OOH CPR by cause and number of survivors

Cause of out-of-hospital arrest	Number of patients	Percentage of total OOH CPR	Survivors	Percentage survivors/group
Trauma	45	22	2	4
Drowning	26	13	8	31
At Birth	7	3	4	57
Non-trauma	115	57	15	13
Unknown	8	4	0	0
Total	201		29	

Nine patients (4%) were lost to follow up and we could not assess if they survived and were discharged from hospital. Out of the 192 subjects, 29 patients survived to discharge from hospital (15%). Two of them suffered from severe neurological disability. In eight patients, there was no registration of the indication of OOH CPR, due to missing data (one hospital refused to give data).

Mean age is 5.0 years with a range 0–16.9 years. The first rhythm related to survival until discharge from hospital is shown in Table 2. In the survivor group, all pediatric patients showed return of spontaneous circulation (ROSC) before leaving the incident scene to hospital.

**Table 2:** First rhythm with number of patients related to survival until discharge from hospital

First rhythm	Number of patients	Number of patients who survived until discharge	Percentage (%)
Asystole	130	7	5
Bradycardia	26	6	23
Unknown	14	7	50
Ventricular fibrillation	9	7	78
Ventricular tachycardia without output	1	1	100
Pulseless electrical activity	12	1	8
Sinus tachycardia	9	0	0

HEMS performed intubation in 104 patients, 79 intubations were done by EMS alone and eight by EMS under direct supervision by an HEMS physician. Unsuccessful primary intubations by EMS without supervision occurred in 40 of 79 intubations (51%). In 27 of the 40 unsuccessful intubations, EMS tried to intubate, but failed, another seven tubes were placed intraesophageally, four intrabronchially, and one neurotrauma pediatric patient was intubated after ROSC without additional medication. The overall success rate by EMS was 57 versus 99% for HEMS. Only one intubation done by HEMS failed, because the patient could not be intubated due to rigor mortis, and the team feels responsibility to the parents to carry on for psychosocial reasons.

Intravenous access was successfully achieved in 76 patients, 27 by HEMS versus 41 by EMS (8 unknown). Intraosseal access was achieved in 139 patients, 43 by HEMS versus 33 by EMS (63 Unknown). Intraosseal dislocation (first attempt successful in bone marrow then dislocated) was found in four patients and extraosseal placement in 14 patients (not in bone marrow), all done by EMS.

ALS procedures in the survivor group are shown in Table 3. HEMS did the majority of these procedures. Failures by EMS were extubation by accident (1), intrabronchial intubation (1), leakage due to too small tube size (1), tube intraesophageal (2), and failed intubation [tried to intubate but not succeeded (3)]. In the survivor group, 15 pediatric patients received medication by HEMS not mentioned in the nationwide EMS protocol, such as sedation, relaxation, antibiotics, and medication to regulate blood pressure.

**Table 3:** ALS procedures in survivor group by HEMS and/or EMS

ALS procedure	HEMS	EMS with HEMS supervision	EMS	EMS failure	
Intubation	23	2	10	8	2 not intubated
IO access	10		6	3	
IV access	8		3		
Medication not stated in APLS guidelines	15				

## Discussion

We found a 15% survival rate in OOH CPR of children. Studies in other countries [3–7] show lower survival rates (2.1–11%). A possible explanation is the group of low-risk OOH CPR at birth. The Netherlands has a high number of planned birth delivery at home. In the 90s, 35% of pregnant women had a planned delivery at home. In 2012, still, 20% of pregnant women had a planned delivery at home. Although delivery at home is decreasing, a significant number of pregnant women choose to deliver at home rather than a (planned) delivery in hospital. In other countries, the vast majority of women have planned birth in hospital rather than at home. To compare this study to others, taking out the OOH CPR at birth, the overall survival rate would be 13% instead of 15%. When HEMS became operational, EMS frequently secondary asked for assistance in stabilizing vitally compromised children. After a few years, it became protocol to activate HEMS primarily in vitally compromised children and over the years HEMS gained more and more experience. Nowadays, 21% of all calls are concerning pediatric patients. That could also be an explanation why the survival rate from the early years has gone up to 13%.

In our study, EMS performed less ALS procedures than HEMS. Maybe, EMS would have done more ALS procedures if HEMS took more time to get on scene or was not available. However, in a large number of cases, HEMS arrived later at the scene, but EMS only provided BLS care; in cases, they also should have performed ALS procedures. HEMS did the majority (73%) of ALS procedures in the survivor group of 29 patients. Especially the patients who were intubated not correctly or had a non-working intraosseous access, HEMS could have altered the outcome by emergency correction of the endotracheal tube and/or intraosseous cannulation (Table 3). In addition, intubation done by an HEMS physician was more successful than by an EMS paramedic. Members of HEMS have more training, experience and exposure. Successful intubation in children seems to be a difficult task for EMS paramedics [1, 9, 10]. Bag-mask ventilation is to be preferred to a failed intubation effort, even if bag-mask ventilation is suboptimal [9]. Pediatric airway skills decay quickly after training because of the low-call volume, and endotracheal intubation skills drop off more significantly than bag-mask ventilation skills [11]. HEMS had to perform an emergency correction of the tracheal tube in 11 patients (14%). In the opinion of the authors, this is an unacceptable rate. Because of the high rate of unsuccessful intubations, EMS in The Netherlands should not intubate children.

It is difficult to measure long-term neurological outcome in (very) young children. Only few of them had a prior admission to hospital, which make it difficult to objectively assess a small neurological deficit. Two pediatric patients were obviously severely neurological impaired and scored severe disability based on a modified Pediatric Cerebral Performance Category Scale [12]. After the incident, they remained in a wheelchair and are not able to perform normal activities of daily living. The other 27 survivors are scored in the category normal or no change from baseline or mild disability. There is no previous scoring so it is difficult to set a baseline, but most of the children in this group score conform other children of their age.

All survivors in our study had ROSC before transport to hospital. This could mean that there is time to set up a good BLS and ALS rather than “scoop and run” to hospital.

Prior to our study and prior to 1997 (the start of Rotterdam HEMS), there are no studies available with only EMS and OOH CPR in pediatric patients performed in The Netherlands with outcome known to the authors.

Another finding was that HEMS delivered additional medication, not stated in national EMS protocol to 70 patients. Most of the medication was given after ROSC, for instance, medication to regulate blood pressure (and thus cerebral blood perfusion), sedation, or antibiotics in sepsis.

The retrospective design of this study may be considered as a limitation. In addition, in nine (4%) patients, there were missing data and we could not assess if they lived up to discharge from hospital.

After reviewing all pediatric out-of-hospital cardio pulmonary resuscitations (OOH CPRs), 29 patients (15%) survived until discharge. The Helicopter Emergency Medical Service (HEMS), especially the younger the patients, performed the majority of the advanced life support (ALS) skills, such as intubation, intraosseous, and/or intravenous access, and had less failures than in procedures performed by Emergency Medical Service.

Therefore, we conclude that Helicopter Emergency Medical Service brings essential skills and expertise to the scene of out-of-hospital cardio pulmonary resuscitation in pediatric patients.
